# Complementary computational and experimental evaluation of missense variants in the ROMK potassium channel

**DOI:** 10.1371/journal.pcbi.1007749

**Published:** 2020-04-06

**Authors:** Luca Ponzoni, Nga H. Nguyen, Ivet Bahar, Jeffrey L. Brodsky

**Affiliations:** 1 Department of Computational and Systems Biology, School of Medicine, University of Pittsburgh, Pittsburgh, Pennsylvania, United States of America; 2 Department of Biological Sciences, University of Pittsburgh, Pittsburgh, Pennsylvania, United States of America; Max Planck Institute for Biophysical Chemistry, GERMANY

## Abstract

The renal outer medullary potassium (ROMK) channel is essential for potassium transport in the kidney, and its dysfunction is associated with a salt-wasting disorder known as Bartter syndrome. Despite its physiological significance, we lack a mechanistic understanding of the molecular defects in ROMK underlying most Bartter syndrome-associated mutations. To this end, we employed a ROMK-dependent yeast growth assay and tested single amino acid variants selected by a series of computational tools representative of different approaches to predict each variants’ pathogenicity. In one approach, we used *in silico* saturation mutagenesis, i.e. the scanning of all possible single amino acid substitutions at all sequence positions to estimate their impact on function, and then employed a new machine learning classifier known as Rhapsody. We also used two additional tools, EVmutation and Polyphen-2, which permitted us to make consensus predictions on the pathogenicity of single amino acid variants in ROMK. Experimental tests performed for selected mutants in different classes validated the vast majority of our predictions and provided insights into variants implicated in ROMK dysfunction. On a broader scope, our analysis suggests that consolidation of data from complementary computational approaches provides an improved and facile method to predict the severity of an amino acid substitution and may help accelerate the identification of disease-causing mutations in any protein.

## Author summary

As the number of sequenced human genomes rises, a major challenge is to identify which single amino acid variations in a protein affect function and predispose individuals to disease. While predictive algorithms are available for this purpose, a comparative analysis of recently developed algorithms has not been adequately performed, nor is it clear whether combining algorithms would improve predictive power. To this end, we compared the efficacy of three publicly available algorithms and applied the results to Bartter syndrome, a human disease for which numerous poorly-characterized single amino acid variants have been identified and for which there is no cure. *In silico* saturation mutagenesis, i.e., the computational prediction of pathogenesis for every possible amino acid substitution, allowed us to experimentally test predictions by measuring the activity of an ion channel linked to Bartter syndrome. Based on data from blinded experiments, we discovered that Rhapsody and EVmutation successfully predicted deleterious mutations. Moreover, Rhapsody—which takes into account evolutionary as well as structural and dynamic considerations—predicted that >90% of known Bartter syndrome mutations are deleterious. Overall, our data will aid investigators who wish to test single amino acid variants in any protein and aid biomedical researchers who wish to develop hypotheses on the potential severity of genetic variants uncovered from genome databases.

## Introduction

The Renal Outer Medullary Potassium (K) Channel, ROMK, is a member of the Kir family of inwardly rectifying potassium transporters and resides most prominently in two regions of the kidney, the thick ascending loop of Henle and the cortical collecting duct [1]. Members of the seven families of Kir channels are found in every major organ and can be gated by voltage or cyclic nucleotides, whereas others, like ROMK, are constitutively active [2]. Therefore, the levels and activity of ROMK are instead controlled by biosynthesis, transport, or retention at the plasma membrane, as well as by pH, PIP_2_, and protein tyrosine kinases.

**Fig 1:**
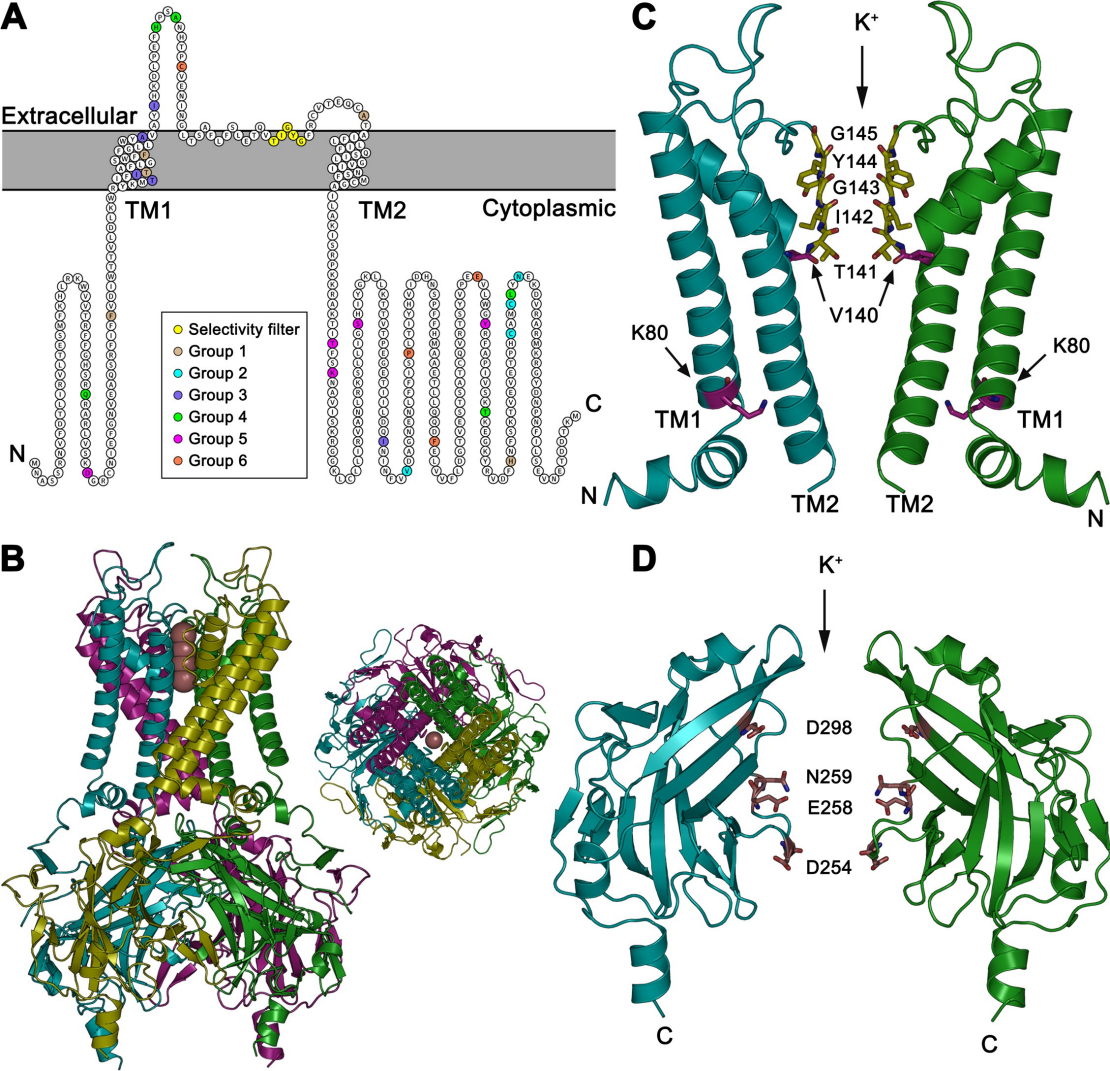
ROMK structure. (A) A linear structural model of the human ROMK1 monomer. Residues important for potassium transport in the pore helix (yellow) and residues tested in this study (Groups 1–6) are highlighted. ROMK1 shares a common tetrameric architecture with other inward rectifying (Kir) potassium channels: two transmembrane (TM) domains TM1 and TM2, a conserved potassium selectivity filter and cytoplasmic N- and C- terminal domains. The linear model was generated using Protter [52] (UniProt accession no. P48048). (B) A ROMK1 homology model (aa 38–365) shows the oligomerization of four ROMK1 subunits to form a functional channel with a central pore through which the potassium ions pass. The homology model was built based on the crystal structure of Kir2.2 (PDB ID 3SPG), which is 47.4% identical to ROMK1 (which is well within the range suitable for accurate comparative modeling). Images were rendered using PyMOL (v2.1.0). (C) The ROMK1 potassium selectivity filter contains the indicated “T_141_IGYG_145_” motif. Main-chain oxygens in this motif line the pore and facilitate potassium entry. V140, which is located between the selectivity filter and the pore-lining helix TM2, contributes to single channel conductance and barium block [28]. In TM1, K80 is important for the interaction between TM1 and TM2 and controls channel gating [5, 34]. (D) The cytoplasmic extended pore includes residues D254, E258, N259, and D298. Panels C and D display portions of two subunits. The other two were removed for visual clarity.

The active plasma membrane resident ROMK species is formed from four identical ~45 kDa polypeptides. Each subunit contains two transmembrane (TM) domains and large cytoplasmic domains at the N- and C-termini, all of which stabilize the tetramer [3]. In turn, the ion-conducting channel is formed at the interface of the four pore or “slide” helices, which connect the two TM domains and face the extracellular space [4] (**Fig 1**). Residues in the second TM domain and in the C-terminus also contribute to potassium transport. Like other membrane proteins that ultimately reside at the plasma membrane, ROMK is initially synthesized on endoplasmic reticulum (ER)-associated ribosomes. ROMK monomer folding and tetramer assembly most likely take place in the ER, which are essential for subsequent trafficking to the cell surface.

As might be anticipated based on the importance of potassium in homeostasis and the broad distribution of Kir channels in the body, amino acid substitutions (i.e., mutations) in these proteins lead to a range of diseases. For example, individuals carrying two defective copies of the gene encoding ROMK, KCNJ1, present with Type II Bartter syndrome, a life-threatening salt-wasting disorder in which children fail to thrive and are afflicted with a range of metabolic, renal, and neurological disorders [5, 6]. Some of the Bartter syndrome-associated mutations compromise ROMK folding in the ER, which leads to the selection and targeting of ROMK for proteasome-dependent degradation [7]. Precisely how these mutations affect ROMK folding and/or assembly is unknown, as are the molecular defects underlying most of the ~40 Bartter syndrome mutations that have been identified [8]. In contrast, individuals with only one mutated copy of KCNJ1 have a lower risk of hypertension and decreased blood pressure [9]. Because nearly all blood pressure lowering drugs exhibit unwanted side-effects, the development of ROMK inhibitors has been actively pursued [10, 11]. A realization of this goal would also benefit from a deeper understanding of ROMK folding and assembly, as well as the conformational dynamics that mediate channel opening.

As a result of alternative splicing, there are actually three ROMK isoforms that contain slightly different N-terminal sequences. These isoforms are expressed in different segments of the kidney nephron, resulting in distinct physiological outcomes [12, 13]. For example, while ROMK2 is localized to the thick ascending limb and contributes to sodium reabsorption, ROMK1 and ROMK3 are responsible for maintaining potassium balance more distally in the nephron [5]. Regardless, as patients with Bartter syndrome type II present with defects in both sodium and potassium regulation [14], all ROMK isoforms almost certainly play a role in the disease.

The 3D structure of human ROMK has not been resolved at the time of writing, but a ROMK homology model (for residues 38 to 365) is deposited in the SWISS-MODEL repository [15], which was built by using the crystal structure of Kir2.2 (PDB ID 3SPG) [16] as a template (see **Fig 1**). Nevertheless, a comprehensive structure-based understanding of ROMK residues that might be susceptible to disease-causing alterations remains unknown. Moreover, it is unknown if gain-of-function mutations within ROMK in individuals might exist. These are predicted to alter salt and water homeostasis and, consistent with the fact that individuals with a single mutated version of ROMK have a lower risk of hypertension, gain-of-function mutations could lead to hypertension.

In recent years, several studies—some accompanied by publicly accessible tools—have supported the idea that structural data, in addition to classical conservation analyses based on multiple sequence alignments (MSAs), provide a means to predict the effect of single amino acid substitutions on biological function [17–20]. We recently showed that a consideration of not only structure but also structural dynamics can further improve the accuracy of these predictions [21]. Based on the importance of ROMK in human health and the growing number of mutations and polymorphisms identified in this channel, we set out to analyze a dataset of single amino acid variants (SAVs) in the human ROMK1 gene (UniProt accession no. P48048). Specifically, we used our new machine learning (ML)-based classifier and the corresponding interface, Rhapsody (http://rhapsody.csb.pitt.edu/), which we recently implemented [22]. The method incorporates dynamics-dependent properties such as local residue flexibility and other allosteric and mechanical features computed by the Elastic Network Model (ENM) analysis of the protein’s structural dynamics [23], in addition to those dependent on sequence and structure, to evaluate the impact of SAVs [21]. Thus far, Rhapsody has been validated [22] on a dataset of ~20,000 labelled human variants to yield state-of-the-art accuracy levels. Previous comparison with dozens of alternative approaches (including a few based on structural data), chosen from amongst more than one hundred currently available methods has demonstrated that Rhapsody’s performance consistently ranked among the highest, based on multiple accuracy metrics [21, 22]. As reported here, we selected a dataset of 33 SAVs in ROMK and examined their impact using Rhapsody and other advanced pathogenicity prediction tools, namely EVmutation [24] and PolyPhen-2 [25]. EVmutation is a coevolution-based algorithm that performs direct coupling analysis of Pfam domains to assess critical interactions, and PolyPhen-2 is a widely used tool that combines conventional MSA analysis techniques with structure-derived properties in a probabilistic classifier. Next, we tested the SAVs on ROMK function in two assays that harness a quantitative measure of potassium-dependent cell growth. Our comparative analysis supports the power of our new platform for *in silico* saturation mutagenesis studies and the utility of consolidating results from multiple tools, while also pointing out the limitations of existing techniques. Our data also highlight key sub-domains in ROMK and additional SAVs that might be linked to disease. More generally, this study serves as a beacon for those who wish to use computationally-guided mutagenesis and predict pathogenesis based on naturally occurring SAVs.

## Results

### *In silico* saturation mutagenesis analysis of ROMK

In this study, we focus on ROMK SAVs motivated by: (i) their potential link to a catastrophic disease, type 2 Bartter syndrome, for which there is no cure, (ii) the fact that the effects of most disease-causing mutations on ROMK function are ill-defined, and (iii) the lack of knowledge on the consequences of numerous SAVs in ROMK, which have been reported in the human genome database (https://www.nhlbiwgs.org/). As noted above, in humans, there are three splice variants of ROMK (ROMK1, ROMK2, and ROMK3) that modestly differ at their N-termini, but because the ROMK isoforms exhibit identical transport activity [12, 26], because mutations in the sequence common to each ROMK variant can cause Bartter syndrome, and because most published studies have utilized ROMK1, our analysis focused on this isoform.

**Fig 2:**
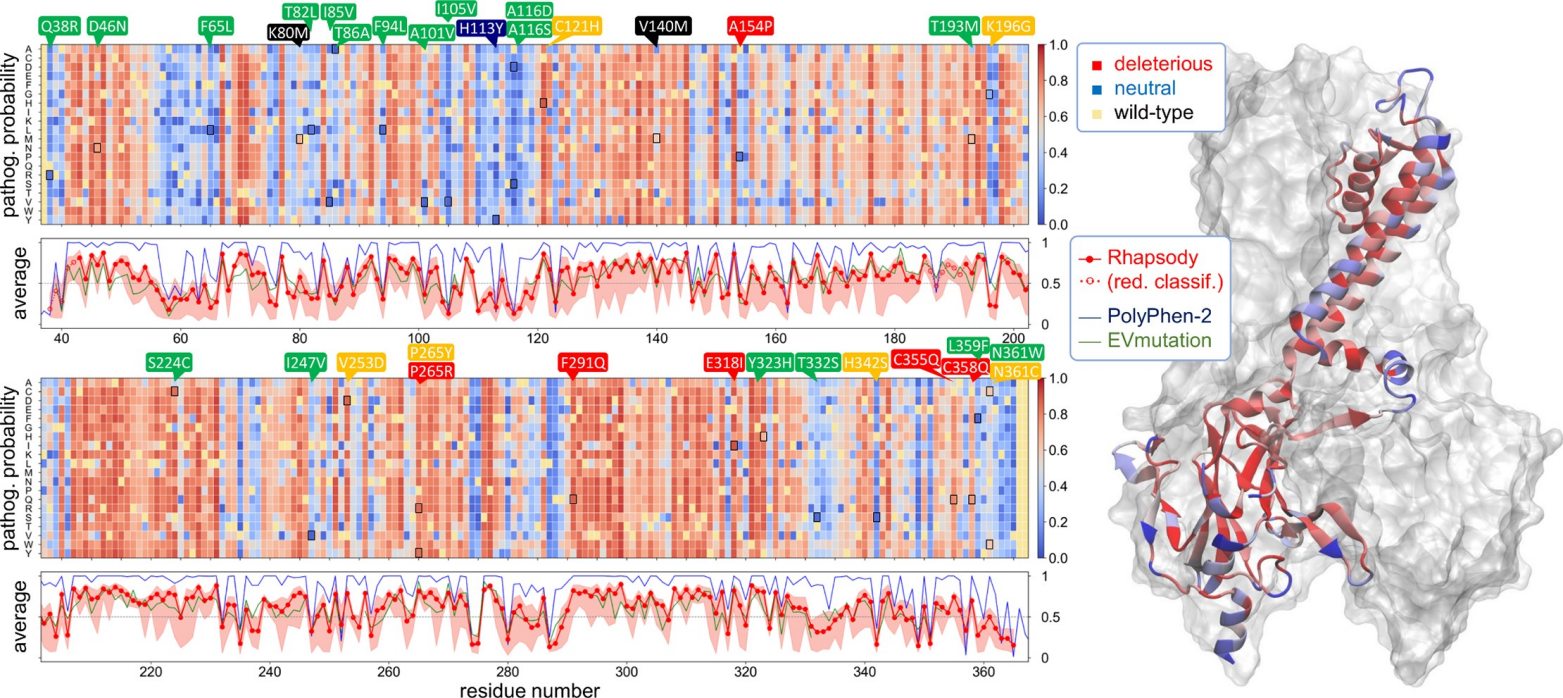
*In silico* saturation mutagenesis analysis of ROMK variants. The predicted pathogenicity probabilities obtained with Rhapsody are shown in a heat map (divided in two parts) with a color code ranging from *red* (deleterious, >0.5) to *blue* (neutral, ≤0.5). *Light yellow* entries correspond to wild-type (WT) amino acids. The *bottom panels* corresponding to each heat map represent the residue-averaged pathogenicities predicted by Rhapsody (*red*), EVmutation (*green*) and PolyPhen-2 (*blue*). The *pink shade* indicates the range of Rhapsody results for the 19 specific substitutions for each residue. The locations of 31 variants selected for experimental testing and two controls are marked by *black squares* on the map, and callouts colored by the measured phenotype, with the same color scheme as in **Figs 3 and 5**. On the right, the residue-averaged predictions from Rhapsody are displayed for a single monomer in the human ROMK1 homology model using the same color code as in the map.

Since the ROMK structure has not been determined, we relied on a homology model [27] for the UniProt sequence P48048 (isoform 1), which includes residues 38 to 365. Next, the full spectrum of computational predictions for all possible variants in the ROMK1 gene (UniProt name KCNJ1_HUMAN, accession no. P48048) was computed using Rhapsody. Importantly, no ROMK1 variant was included in the training dataset to ensure completely unbiased predictions. The results from this *in silico* mutagenesis study are summarized in **Fig 2** with the help of a heat map (presented into two parts, for clarity). Each entry in the heat map is color-coded by the variant’s predicted “pathogenicity probability”, ranging from 0 (neutral, *blue*) to 1 (deleterious, *red*) for all residues (*abscissa*) and all possible substitutions (*ordinate*). This initial analysis highlights hotspots and sub-domains in which SAVs might exhibit more significant effects on protein structure and function (columns predominantly in *red*).

The *right panel* in **Fig 2** displays the structure used to derive dynamics-based features. One of the relevant subunits is shown in ribbon representation color-coded by the residue-averaged pathogenicity probability values. The residue-averaged pathogenicities are plotted in the panels under the heat maps. This plot indicates the general sensitivity of a given residue to any mutation as predicted by Rhapsody (*red curve*), EVmutation (*green*) and PolyPhen-2 (*blue*). EVmutation [24] takes into account the full landscape of amino acid (co)evolution coupled with the mutation site, which has proven to yield highly accurate results. PolyPhen-2 [17, 25] uses sequence conservation, sequence annotations, and static structural properties in a supervised naïve Bayes classifier. These tools were chosen because of the complementarity of the underlying methods and features. A recent comparative study of a comprehensive dataset of ~20,000 labelled human missense variants [22] showed that Rhapsody outperformed EVmutation, which in turn yielded more accurate predictions than PolyPhen-2, although the coverage of the latter was much wider since multiple sequence alignment data from Pfam was unnecessary. For this particular analysis of ROMK variants, PolyPhen-2 was able to return predictions for the complete set of SAVs, while Rhapsody and EVmutation had respective coverages of 84% and 77%. Remarkably, PolyPhen-2 recognized as potentially deleterious the majority of variants (~77%), while Rhapsody and EVmutation provided more balanced predictions (~56% and ~49% “deleterious”, respectively).

### Comparisons from multiple tools help categorize single amino acid variants

**Fig 3:**
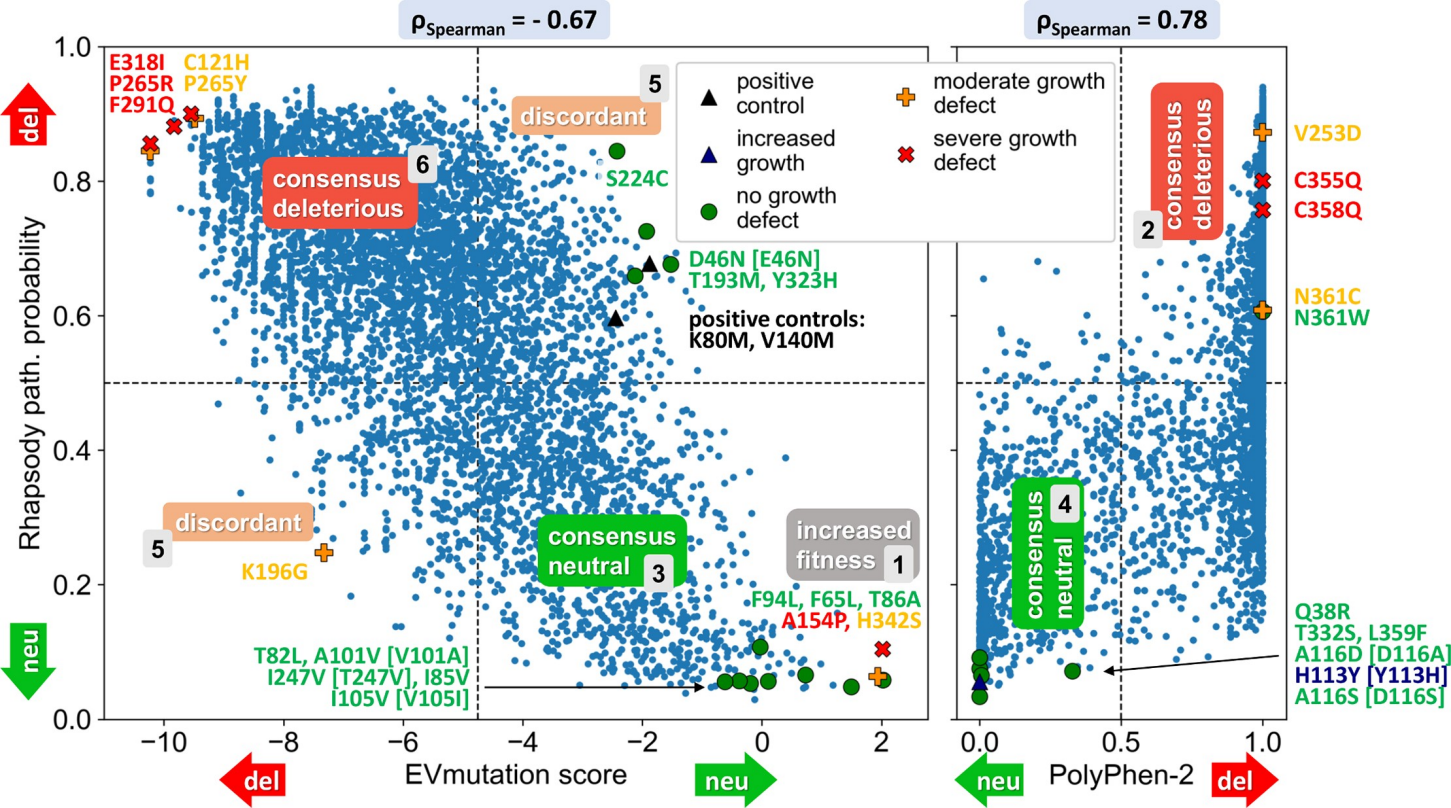
Scatter plots comparing computational predictions and identifying consensus and discordant data. The two scatter plots compare the predictions from Rhapsody and EVmutation (*left*) and those from Rhapsody and PolyPhen-2 (*right*), allowing the outputs to be grouped in three different categories: consensus neutral, consensus deleterious, and discordant, as indicated by the labels. Cutoff values between neutral/deleterious predictions for each method are represented by dashed lines. Note that EVmutation’s ΔE score anticorrelates with the expected pathogenicity of variants. The 31 variants selected for experimental validation and two controls (see text, **Fig 5** and **S1 Table**) are labelled in the two plots and marked by different symbols and colors based on the experimentally observed phenotypes. Results for those variants which cannot be evaluated using EVmutation, due to the absence of a suitable Pfam domain and/or MSA, are shown in the right plot only, with abscissa values based on PolyPhen-2 scores. Labels written in square brackets refer to rat ROMK1 variants that were experimentally tested after substituting for the counterparts in the human sequence.

We next compared the results obtained by EVmutation and PolyPhen-2 for ROMK1 (**Fig 3**). The scatter plot on the *left* compares predictions obtained from Rhapsody and EVmutation for all amino acid substitutions. Although both methods extract conservation and coevolutionary features from Pfam protein families, Rhapsody also includes (and mostly relies on) protein structure and intrinsic conformational dynamics. In addition, the pathogenicity probability returned by Rhapsody allows one to classify variants with a value greater than 0.5 as deleterious. EVmutation, on the other hand, does not provide this classification. Instead, it provides a score representing the change in evolutionary statistical energy (ΔE) between the mutant and wild-type (WT) sequence via an epistatic model that captures pairwise couplings. Larger negative ΔE values indicate a lower probability for the sequence to tolerate a given amino acid substitution, whereas values close to 0 indicate an evolutionary fitness comparable to that of the WT sequence. Positive values, albeit rare, might suggest increased fitness, increased activity, or a potential “gain-of-function” effect. A cutoff for deducing pathogenicity classes (neutral or deleterious) from ΔE values was defined here by optimizing its performance in our training dataset (see **Materials and Methods** for more details). A similar scatter plot between predictions uncovered by Rhapsody and PolyPhen-2 is shown in the *right* panel.

The results presented in **Fig 3** show that Rhapsody and EVmutation exhibit reasonable agreement despite their distinct criteria and methods. The level of agreement between Rhapsody’s predicted pathogenicity probability and EVmutation’s *negative* score (-ΔE) is quantified by Spearman’s rank-order correlation to be 0.67. An integrated version of Rhapsody that incorporates the EVmutation score as an additional ML feature—a strategy that was found to slightly increase overall accuracy [22]—raises the (absolute) correlation to 0.86 (see **S1 Fig**). Rhapsody and PolyPhen-2, on the other hand, exhibit a correlation of 0.78, which could be attributed to similar features of the training algorithms (e.g., sequence conservation, position-specific residue substitution, and solvent-accessible surface area). PolyPhen-2 exhibits a pronounced bimodal distribution, with most predictions located at the two ends of the [0, 1] interval (**S3 Fig**). Together, these data indicate that a direct comparison between predictions obtained by orthogonal approaches helps identify SAVs that share concordant predictions (*diagonal blocks* labeled as “consensus deleterious” and “consensus neutral” in **Fig 3**). Similarly, a comparison of the methods allows for the recognition of “discordant” mutations based on predicted sensitivities/pathogenicities (off-diagonal blocks).

### Experimental evaluation of single amino acid variants reveals the utility of predictive data consolidation

A comparative analysis of computational data from Rhapsody, EVmutation, and PolyPhen-2 (**Fig 3**) defined the following six categories of predictions, referred to as Groups 1–6: consensus neutral (group 3), consensus deleterious (group 6), and discordant (group 5) between Rhapsody and EVmutation predictions (in different quadrants of the scatter plot, in **Fig 3**, *left*); consensually predicted (deleterious and neutral, labeled respectively as groups 2 and 4) by Rhapsody and PolyPhen-2, only (**Fig 3**, *right*); and a subset of consensus neutral predictions (group 1) between Rhapsody and EVmutation which showed a positive ΔE according to EVmutation. This category might indicate an increased fitness or a gain-of-function of a mutant protein compared to a WT protein. On the contrary, the mutant might be too stable/ordered and suppress protein activity by precluding WT dynamics (also see **Discussion**).

To assess whether the predicted consequences of select variants exhibited the expected phenotypes, 31 ROMK variants spanning the six groups (see **Figs 1A**, **2** and **3** and **S1 Table**) were chosen. The SAVs were selected based on: (i) the relatively high confidence of computational predictions, (ii) whether a variant exists at the same position in the human polymorphism database (see **Materials and Methods**), and might thus be disease-relevant, and (iii) if the variant was isolated in other genetic screens for ROMK (see below). For this study, rat ROMK1 was used based on its established activity [6, 28]. Because select human variants corresponded to the WT sequence in rat (namely A101V, I105V, H113Y and A116D), rat-to-human versions were tested instead (i.e., V101A, V105I, Y113H and D116A). For three other SAVs with a different WT amino acid in the rat sequence (D46N, A116S, I247V), the counterparts in rat were considered (i.e. E46N, D116S, T247V). Of note, rat and human ROMK are 91% identical at the amino acid level. Finally, we included two variants (K80M and V140M), which were not counted amongst the 31 variants and which appeared to exhibit increased activity or a gain-of-function phenotype when compared to WT ROMK in pilot studies. These alleles were selected because the K80M allele exhibits increased ROMK channel activity in yeast lacking endogenous potassium transporters [29], while the V140M mutation was identified in this screen. These alleles therefore served as positive controls for our experimental analysis. In total, we created 33 variants, which are labeled in **Figs 1–3** and **S1 Table**. Importantly, blinded experiments were performed, such that the experimentalists were unaware of the group (1 through 6) to which the mutant belonged until all data were obtained.

**Fig 4:**
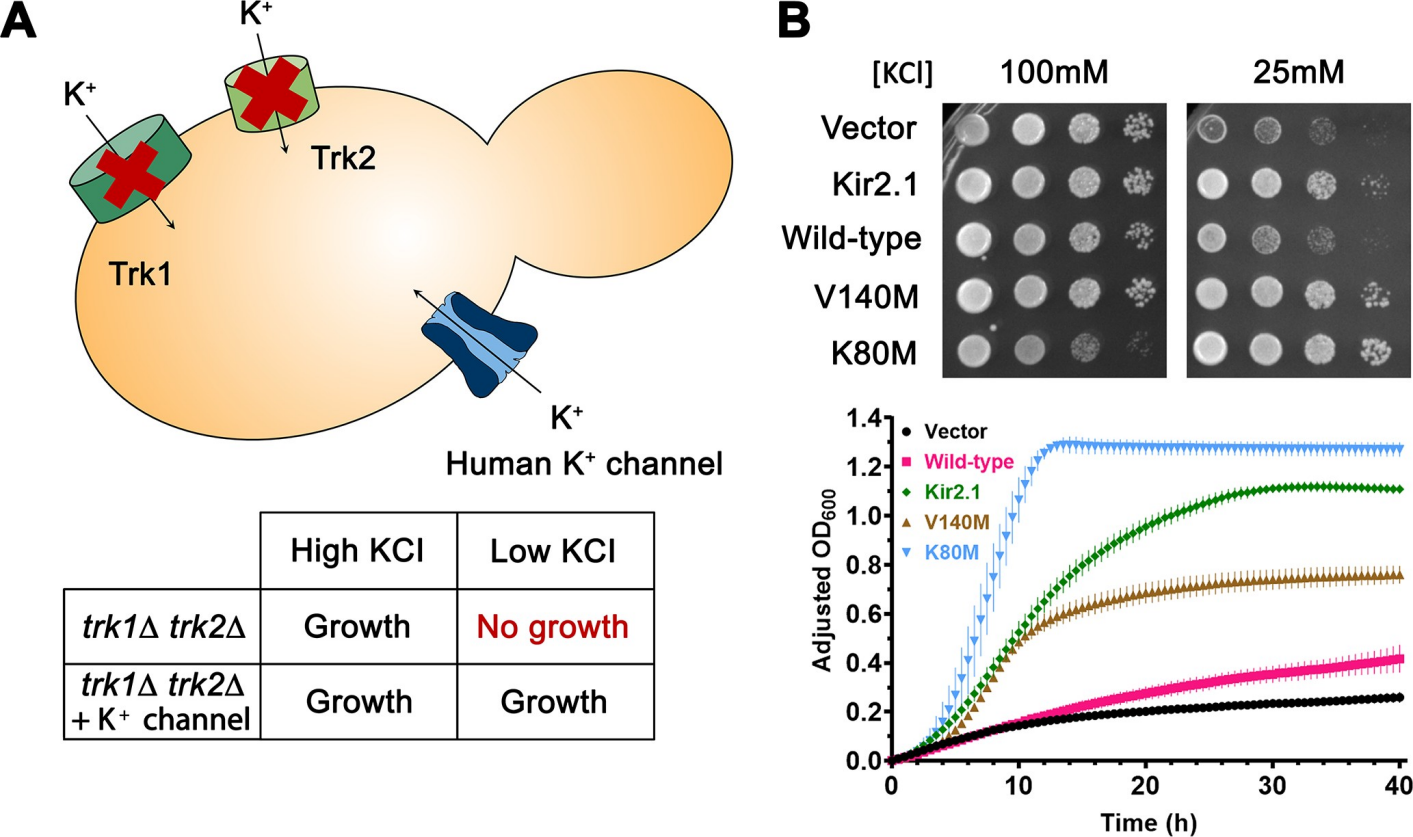
A yeast-based assay to assess the activity of a heterologously expressed potassium channel. (A) Schematic of the yeast-based assay. A yeast strain lacking its endogenous potassium transporters, Trk1 and Trk2, is unable to grow on medium containing low potassium but can be rescued by the expression of a human potassium channel. (B) Controls for yeast viability assays on solid (*top panel*) and in liquid (*bottom panel*) medium. Yeast cultures were transformed with an empty expression vector as a negative control, or with a plasmid expressing Kir2.1, WT ROMK1, or ROMK1 with the V140M or K80M mutation. Kir2.1, ROMK1 V140M and K80M were used as controls, and V140M and K80M exhibit increased growth compared to cells expressing the WT channel. For the viability assay on solid medium (*top panel*), saturated overnight cultures of yeast were serially diluted and spotted on SC-Leu medium supplemented with dextrose and containing the indicated concentration of potassium. Plates shown were imaged after a two-day incubation at 30°C and are representative of three independent experiments. For the viability assay in liquid medium (*bottom panel*), yeast grown overnight to saturation were diluted to an OD_600_ of 0.20 with medium supplemented with 25mM KCl. OD_600_ readings were recorded, normalized to wells containing only medium, and these values were subtracted from the reading at t = 0 (see Materials and Methods). Graphs were made using GraphPad Prism (v8. 1. 2), and data represent results from four independent experiments (n = 2–3 each), ± the range of the data. The growth of K80M on 100mM KCl is reduced because K80M enhances potassium uptake and intracellular potassium concentration, which is toxic.

To assay the function of each ROMK variant, we employed a quantitative yeast-based screen in which the relative growth of a yeast strain expressing WT or mutated ROMK can be measured [7, 30]. The assay takes advantage of the fact that yeast growth on low potassium medium requires the exogenous expression of a potassium channel. Specifically, if the yeast cells lack their endogenous potassium channels, which are known as Trk1 and Trk2, they are inviable unless an active potassium channel is expressed in place of Trk1 and Trk2 [31] (**Fig 4A**). Yeast growth on low potassium can then be assayed using complementary screens on both solid medium as well as liquid medium.

**Fig 5:**
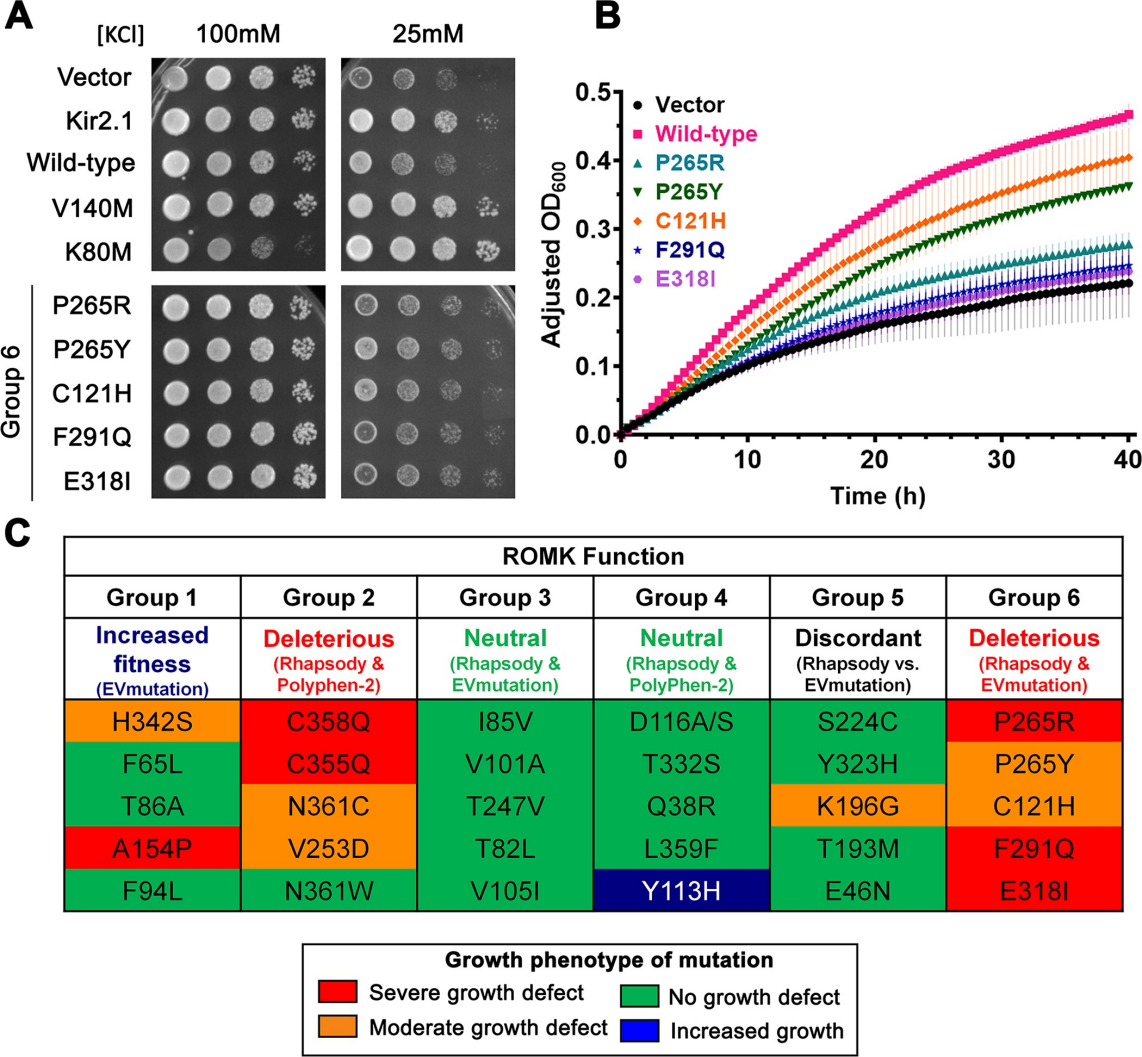
Growth phenotypes of trk1Δtrk2Δ yeast expressing the ROMK1 variants. Representative yeast viability assays with control strains and the ROMK1 variants in group 6 were performed on (A) solid and (B) liquid medium. (A) Ten-fold dilutions of overnight, saturated cultures of yeast were inoculated on medium as described in **Fig 4**. Images were taken after two days of incubation at 30°C. (B) Saturated yeast cultures were diluted to a starting OD600 of 0.2 with assay medium supplemented with 25mM KCl and grown at 30°C. OD600 readings were recorded and data were standardized as described in the Materials and Methods and in **Fig 4**. Data represent results from two independent experiments (n = 2–3 each), ± the range of the data. (C) Table summarizing the growth phenotypes of the six ROMK1 groups described in the text. The predicted consequence of each group is denoted. Growth phenotypes were obtained in a blinded fashion, compared to the growth of *trk1*Δ *trk2*Δ yeast expressing WT ROMK1, and the results are color-coded: Red denotes a severe growth defect, orange denotes a moderate growth defect, green denotes no growth defect (WT-like), and blue denotes a slight increase in growth compared to the WT control. These classifications were performed by visual inspection. For example, as shown in **Fig 5A and 5B**, the P265R variant exhibited levels of growth that matched the vector control (i.e., the errors overlapped in **Fig 5B**), and hence this mutation was designated “severe growth defect” in part (C). In contrast, the P265Y variant exhibited levels of growth that were intermediate to that of the vector control and “Wild-type” (**Fig 5B**). Hence, this mutation was designated “moderate growth defect” in part (C).

As shown in **Fig 4B**, we first determined that yeast expressing WT ROMK1 or Kir2.1, another Kir channel, grew significantly better than yeast containing only the vector control on either low potassium solid medium (*top*) or liquid medium (*bottom*), as expected [7, 30, 32]. We also found that the K80M and V140M alleles exhibited a gain-of-function phenotype, as they grew better than yeast expressing WT ROMK1. Next, we assayed the growth of each of the 31 selected ROMK alleles under identical conditions and compared the results to the WT and K80M and V140M controls. An example of the raw data from this analysis for the SAVs in group 6 are shown in **Fig 5A and 5B**.

In **Fig 5C,** we present the overall results for each of the six groups defined by computations, as described above. These groups represent the six categories of outcomes from comparative analyses of pairs of methods, illustrated in **Fig 3**. The experimental observations are classified into four phenotypes, namely “severe growth defect”, “moderate growth defect”, “no growth defect”, and “increased growth”. To enable a comparison with predictions, the mutations that lead to the first two phenotypes are considered deleterious and the third is neutral. The fourth, a single case (Y113H), is unique (see below). The entries in the table are colored *red*, *orange*, *green and blue* to designate the four respective phenotypes that were experimentally observed. Comparison between experiments and computations shows that Rhapsody and EVmutation correctly classify most of the consensus variants (13 out of 15 variants in groups/columns 1, 3 and 6; see *left panel* of **Fig 3** and last column in **S1 Table**). Rhapsody additionally predicted the correct outcome for 9 out of 10 variants in groups 2 and 4 that were analyzed by Rhapsody and Polyphen-2. Note that these could not be analyzed by EVmutation, due to the limitations of the method (on the *right panel* in **Fig 3,** also see **S1 Table**). On the other hand, EVmutation yielded more accurate results for the five variants with moderate or no growth defect for which the two tools provided discordant predictions (group 5, *top right* and *bottom left* quadrants in **Fig 3**, *left plot*). Such differences are mitigated when the EVmutation score is incorporated in Rhapsody’s classifier as an additional feature (see **S1 Fig**, **S1** and **S2 Tables**). More quantitative accuracy estimates for the three sets of computational predictions, such as ROC curves, Area Under ROC curves and Matthews correlation coefficient, are reported in **S2 Table** and **S2 Fig**. Together, these data highlight the utility of consolidating the predictions from multiple complementary tools, to accurately predict the biological impact of designed or naturally occurring SAVs.

### Structural and mechanistic aspects of ROMK conductance

We found that weakly increased growth—or a putative gain-of-function phenotype—was only observed in a single newly created mutant (Y113H). This phenomenon, as well as that exhibited by the two positive controls, K80M and V140M, might arise from improved channel conductance, improved folding in the ER, and/or facilitated trafficking through the secretory pathway. Intriguingly, Rhapsody instead predicts that K80M and V140M are deleterious, or more formally, that there is a change-of-function. Consequently, this prediction necessitates further scrutiny in the light of protein structure and dynamics. In fact, the V140M substitution might affect the T_141_IGYG selectivity filter as mutations at this position impact channel gating [33]. Likewise, K80 is known to regulate channel gating, and mutations in two neighboring amino acids, Y79 and A177 (the latter of which forms a hydrogen bond with K80), are associated with Bartter syndrome [5, 34]. Therefore, our computational prediction that these residues should be deleterious is in accord with these earlier observations, but not with the observed gain-of-function phenotype.

**Fig 6:**
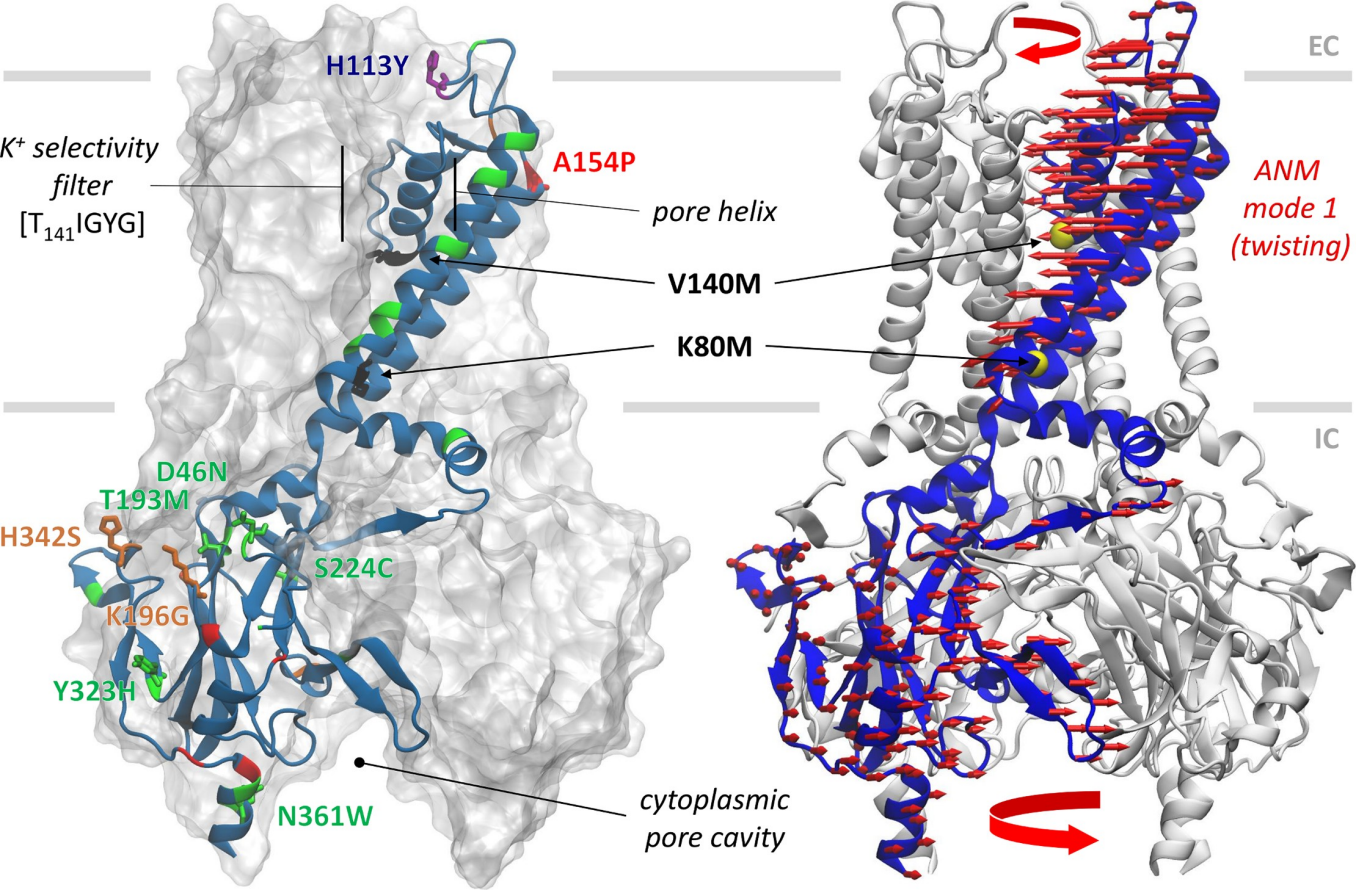
Tested variants on the 3D structure of ROMK. *Left*, residues associated with variants tested experimentally are color-coded on the ribbon representation according to their measured phenotypes, as in Fig 3. *Right*, the dominant twisting deformation computed from an elastic network model of the tetramer is indicated by *red arrows*. K80 is positioned near the interface between the two oppositely rotating (transmembrane and cytoplasmic) regions.

Based on its location in the tetramer (**Fig 6**), we instead hypothesize a role for K80 in defining a dominant mode of channel deformation, as revealed by the first mode of collective motion predicted by the Anisotropic Network Model (ANM) [35]. Information on modes of motions, and in particular, the soft modes that are usually relevant to function, can be readily obtained and visualized using the ANM-based online tools, such as DynOmics [36]. Specifically, K80 is located close to a hinge region at the interface between the transmembrane and cytoplasmic portions of the ROMK tetramer, which undergo a cooperative twisting motion in opposite directions in this particular mode. This is also reflected in the low mean-square-fluctuation (MSF) in the spatial position of that residue, which is one of the features in the Rhapsody classifier (**S4 Fig**). Therefore, we suggest that Rhapsody captures the consequences of a single point mutation on the overall conformational mechanics of the channel, which might elude sequence-based approaches.

In contrast, the isolation of Y113H as a weak gain-of-function SAV is consistent with a previous observation that Y113C, which was isolated in a random mutagenesis screen, conferred heightened ROMK activity in a yeast screen [29]. The mutation allowed for increased potassium transport at low pH, which reflects the acidic environment found in a yeast cell. An analogous interpretation can be made for H342S (moderate growth defect) and A154P (severe growth defect) in group 1 (increased fitness). For these two variants, EVmutation estimates a significantly larger ΔE with respect to the WT amino acid (ΔE = 0), which is described as increased fitness. In this case, the increased fitness or stability somehow suppresses channel function. Examination of the location of these residues (**Fig 6**) shows that they are both located on the protein surface. Moreover, they are predicted to tolerate mutations due to their exposed surface area. However, the replacement of alanine by proline may induce rigidification (in addition to its helix-breaking propensity). The resulting increased stability may then interfere with the conformational adaptability at the extracellular vestibule, which mediates potassium entrance, resulting in the reduced growth rates that were measured experimentally. This type of amino acid-specific conformational mechanics is not incorporated in the training algorithm of Rhapsody. This may explain its inability to detect such potentially deleterious effects. Together, we suggest that the descriptor “change-in-function” is more informative compared to the conventional designation of deleterious, which is adopted by Rhapsody. Likewise, both extreme negative and positive EVmutation ΔE scores could be associated with a change-of-function (i.e., a gain- or loss-of-function), which in turn may manifest as an abnormal phenotype.

### Rhapsody predicts most Bartter syndrome-associated mutations

To date, ~40 mutations in ROMK are known to be associated with Bartter syndrome. With the ever-increasing availability of expanded human genome sequence information, it is imperative that the physiological impact of SAVs can be predicted. Therefore, we examined whether Rhapsody can predict a deleterious phenotype for a set of 34 known Bartter syndrome-associated amino acid mutations that reside in the coding sequence. Based on the definition of a pathogenesis probability of >0.5 as being considered deleterious (**Fig 2**) [20], we found that Rhapsody yielded a pathogenicity score of >0.50 for 29/34 of the mutations, leaving only five variants as being predicted to be neutral (**Table 1**; also see **S5 Fig** for a scatter plot of the Rhapsody predictions). However, among these five, two are classified as “benign” in the clinical variant database [37, 38]. Thus, Rhapsody correctly predicts 31/34 (91%) of the established phenotypes associated with SAVs in the gene encoding ROMK. These data demonstrate the power of Rhapsody as a platform to assess whether any variant in ROMK might be linked to pathogenesis. In the future, it will be imperative to apply this analysis to other monogenic diseases for which facile assays can be used to detect deleterious phenotypes.

**Table 1:** List of known mutations associated with Bartter syndrome and their computationally predicted classification.

mutation	comp. prediction	mutation	comp. prediction
C49Y	**deleterious**	R188C	**deleterious**
I51T	**deleterious**	R188H	**deleterious**
T71M	**deleterious**	A198T	**deleterious**
V72E	**deleterious**	L209F	**deleterious**
D74Y	**deleterious**	A214V	**deleterious**
Y79H	neutral	S219R	**deleterious**
T86A*	neutral*	L220F	**deleterious**
F95S	**deleterious**	G228E	**deleterious**
K107E	neutral	A306T	**deleterious**
D108H	**deleterious**	R311W	**deleterious**
V122E	**deleterious**	S313C	**deleterious**
N124K	**deleterious**	Y314C	**deleterious**
I142T	**deleterious**	L320P	**deleterious**
A156V	**deleterious**	R324L	neutral
G167E	**deleterious**	F325C	**deleterious**
A177T	**deleterious**	P327L	**deleterious**
P185S	**deleterious**	M357T*	neutral*

Amino acid coordinates are numbered according to their position in human ROMK1. Correct predictions are highlighted in red boldface. Out of 34 SAVs reported in the literature to be associated with Bartter syndrome, 29 are correctly predicted by Rhapsody to be deleterious, while 5 are predicted to be neutral. In this group, two variants (T86A and M357T, marked by *) are classified as benign or likely benign in the human clinical variant database. See Materials and Methods for further details. For a distribution of the predicted scores, refer to S5 Fig.

## Discussion

In this paper, we describe a thorough analysis of novel and known SAVs in ROMK1 using an integrated computational and experimental approach that utilizes *in silico* scanning of the effects of all possible mutations as well as functional tests of selected candidates. We also interpret the effects of some of these mutations based on *a priori* computational predictions. More specifically, we demonstrate our ability to obtain reliable predictions on the effects of variants by examining consensus results between complementary computational approaches that derive from unique methodologies. In turn, the study of misclassified variants and differences in computational predictions between individual tools can be used to improve their performance. For instance, from the analysis of control variants that induce a noticeable increase in growth in the yeast assay, we hypothesize that Rhapsody—a prediction algorithm we recently introduced—captures the effects of a variant on the conformational dynamics of a potassium channel. Future work will be used to expand our predictions to encompass a broader range of proteins whose functions can be quantitatively screened in a facile manner.

Based on our data, we also propose a new interpretation of the output from EVmutation. This predictive tool showed remarkable success in recent studies and relies exclusively on the analysis of covariation patterns in multiple sequence alignments to explain experimental observations. EVmutation returns a score, in the form of a statistical energy difference (ΔE), that assesses the pathogenicity of a variant by estimating the reduced fitness of a mutation compared to the WT protein. Based on our experimental results, we hypothesize that an *increment* in fitness might predict a negative phenotype as well. For example, increased fitness may lead to increased protein stability, which can compromise critical conformational changes required for function, or may impact protein function in a specific context, i.e., one that applies to altered environmental conditions. In this respect, it is interesting to note that a recent deep mutational scanning study indicated that reduced solubility accompanies increased fitness (or increased stability) [39]. The adverse effect of increased stability on protein solubility was further corroborated by a systematic computational study [40]. Clearly, the exploration of protein fitness landscapes is a major area of research [41, 42], and a critical assessment of the dynamic consequences of increased stability may assist in designing mutants with increased fitness that also retain the solubility of the WT protein.

We further suggest that new criteria might be needed to interpret results from mutagenesis studies and comparisons with computational predictions. A binary classification of variants’ effects into “neutral” and “change-of-function” might more adequately capture deviations in measured phenotypes from WT levels. In fact, it might not be obvious in some cases if deviations from WT should be attributed to a decreased or increased activity of the target protein, or to other sources, such as modified expression due to a change in protein stability. The same reasoning should be applied to the interpretation of computational predictions.

Finally, the integration of Rhapsody and EVmutation, as distinguished here, is a powerful approach for accurate evaluation of the effects of missense variants. We note, however, that a ROMK homology model was constructed, and a future systematic analysis of the limitations of using homology models, as opposed to experimentally resolved structures, could assess possible limitations in prediction accuracy. More generally, the evaluation of SAVs by meta-predictors that utilize multiple tools became a common practice in recent years, with increased computing resources and advances in ML algorithms. Yet, conflicting data from multiple tools often limits the value of these predictions as users may lack a deep understanding of the molecular (chemical or mechanistic) or cellular bases of the predictions. In contrast, Rhapsody provides detailed (residue-level) information on biophysical, biochemical, and evolutionary features underlying the predictions, and as such offers the possibility of making structure- and dynamics-based interpretations. Furthermore, the feedback loop from experimental validations, as illustrated here for ROMK, appears to be essential to efficiently characterize the impact of mutations and guide further method development. Indeed, the ability of Rhapsody to confirm the deleterious/pathogenic effects of 91% Bartter syndrome-associated mutations (**Table 1**) underscores the utility of this platform, one that will prove valuable as the number of known monogenic diseases rises.

## Materials and methods

### Computational prediction tools

The Rhapsody prediction tool consists of a random forest classifier that combines sequence- (conservation), structure-, and dynamics-based features associated with a given amino acid substitution and is trained over a comprehensive dataset of labelled human missense variants [21]. Dynamical features include: mean-square fluctuations of the residue at the mutation site, which estimates local conformational flexibility; perturbation-response scanning [43] effectiveness/sensitivity, accounting for potential allosteric responses involving the mutation site; and the mechanical stiffness [44] at the sequence position of the mutated residue. These properties are computed from ENM representations of protein structures, that describe inter-residue contact topology in a compact and computationally-efficient format that lends itself to a unique analytical solution for each structure [35]. The algorithm was recently upgraded to include coevolutionary features calculated on conserved Pfam domains, and the training dataset was further expanded and refined [22]. The latter combines annotated human variants from several publicly available datasets (Humvar, ExoVar, predictSNP, VariBench, SwissVar, Uniprot’s Humsavar and ClinVar) and currently contains about 20,000 variants from about 2800 unique PDB chains with at least 150 residues. An integrated classification scheme has also been developed that incorporates EVmutation’s epistatic score (ΔE) as an additional feature of the random forest classifier (“*Rhapsody+EVmutation*” column in **S1 Table**). For the current study, structure-based properties were derived from a homology model of the human ROMK channel retrieved from the SWISS-MODEL repository. EVmutation precomputed scores were converted into binary classes by defining a cutoff (ΔE = -4.75) that maximizes the Youden’s index of the ROC curve computed on Rhapsody’s training examples. A Python Jupyter notebook with instructions for replicating the computational analysis can be viewed and downloaded from the Rhapsody website (rhapsody.csb.pitt.edu) in the section "tutorials/6-Application_to_ROMK/". The complete list of precomputed predictions, in a computer-parseable format (Excel file), can be found in the same location at "data/precomputed_predictions.xlsx".

### Plasmid construction

Rat ROMK1 was amplified from the pSPORT1-ROMK1 vector [45] and inserted into the pRS415TEF1 expression vector [46] using SmaI and XhoI. The cut insert and vector were purified, phosphatase treated, and ligated as described [7]. Point mutations (K80M, V140M, and each of the SAVs analyzed in this study that resided in the ROMK coding region were introduced into the resulting pRS415TEF1-ROMK1 vector using two-step overlap extension PCR mutagenesis [47]. For mutations corresponding to known human variants, we identified polymorphisms available in the Trans-Omics for Precision Medicine program, which provides a polymorphism database of ~149,000 individuals with heart, lung, blood and sleep disorders. The DNA sequences of all variants in the DNA inserts were confirmed by Sanger sequencing (GENEWIZ, NJ). All primers used in this study are listed in **S3 Table**.

### Yeast strain and growth conditions

A *Saccharomyces cerevisiae* strain lacking the Trk1 and Trk2 potassium transporters (*MATα his3*Δ *leu2*Δ *ura3*Δ *trk1*Δ::*URA3 trk2*Δ::*NATMX can1*Δ::*STE2pr-HIS3* [32]) was used to assay the ability of each ROMK1 variant to restore growth on low potassium medium. Plasmids were transformed into yeast via the standard lithium-acetate method [48] and yeast were grown at 30°C in liquid or solid synthetic complete (SC) medium lacking Leu but supplemented with a final concentration of 2% dextrose. The SC medium contained monosodium glutamate as the main nitrogen source and was buffered to pH 4.5 with MES and supplemented with the indicated amount of KCl, as described [30]. Due to the presence of residual potassium in the agar (VWR, PA), each plate contains 7–10 mM KCl, even in the absence of added potassium [49, 50].

### Yeast viability assays

For serial dilutions growth assays on solid medium, cultures were grown overnight at 30°C in SC-Leu medium supplemented with 100mM KCl. Saturated cultures were diluted 10-fold four times in a standard 96-well plate and inoculated onto SC-Leu medium supplemented with 100 mM and 25 mM KCl using a 48-pin replica plater (Sigma, MO). Plates were incubated at 30°C and imaged after two or three days with the Bio-Rad ChemiDoc XRS+ imaging system.

For growth assays in liquid medium, saturated overnight cultures were diluted to a starting concentration of approximately 2 x 10^6^ cells/ml [50] with SC-Leu medium supplemented with 25 mM KCl in a 96-well plate. Plates were covered with a Breathe-Easy gas permeable membrane (Diversified Biotech, MA), and cell density readings were recorded using the Cytation 5 plate reader (BioTek, VT) every 30 min for 40 hrs with constant shaking (559 cycles per min) at 30°C.

### Selection of disease-associated variants

A list of mutations associated with Bartter syndrome was compiled from a clinical variant database [37, 38] and the literature [8, 51]. In the clinical variant database, only missense mutations known to be associated with Bartter syndrome patients were selected. Furthermore, two variants near the N-terminus were omitted due to lack of structure in the homology model we used, which was built based on the crystal structure of Kir2.2 (PDB ID 3SPG).

## Supporting information

S1 Table Experimental results and computational predictions for selected ROMK variants. The 33 ROMK variants tested experimentally are classified into 4 categories (see last column), based on the measured phenotype: “increased growth” (INC), “no growth defect” (NGD), “modest growth defect” (MGD), “severe growth defect” (SGD). Same color scheme is adopted in Fig 5. “Rhapsody+EVmutation” refers to a combined version of the Rhapsody classifier where EVmutation epistatic score has been included as an additional feature. Computationally predicted classes refer to the clusters of variants defined in Fig 3.
(PDF)

S2 Table Accuracy of predictions for 33 ROMK variants tested experimentally, using different metrics. Several accuracy estimates over the set of 33 ROMK variants tested experimentally are listed for each method. As explained in the text, there is no unequivocal interpretation of variants displaying “increased growth”. So, we provided measurements obtained by considering them neutral or deleterious, or after excluding them (indicated by ‘-’ label). Since EVmutation and “Rhapsody+EVmutation” classifiers have missing predictions, for the sake of comparison, we also computed accuracy estimates for Rhapsody and PolyPhen-2 on the same subset of variants (*). In parentheses, we show the bootstrapped mean and standard deviations values.
(PDF)

S3 Table Primers used in this study. (PDF)

S1 Fig Scatter plot comparing the EVmutation and Rhapsody+EVmutation predictions. This figure has the same format as **Fig 3**, but the y-axis is replaced by predictions from the combined Rhapsody classification scheme that incorporates EVmutation’s epistatic scores directly into the Random Forest training algorithm of the classifier, as an additional feature. See **Materials and Methods** for additional information.
(TIF)

S2 Fig ROC curves for computational predictions of tested variants. EVmutation and “Rhapsody+EVmutation” classifiers (*dashed lines*) only return predictions for 22 out of 33 variants. For the sake of comparison, as in **S2 Table**, we also show ROC curves for Rhapsody and PolyPhen-2 predictions on the same subset of variants (*, *dotted lines*). Variants displaying “increased growth” (INC) phenotype have been excluded (*left* column) or included as “neutral” or “deleterious” (*center* and *right* columns, respectively).
(TIF)

S3 Fig Distribution of results (pathogenicity probabilities or scores) from three different tools for pathogenicity prediction applied to ROMK. Normalized histograms are displayed using the data presented in **Figs 2** and **3**.
(TIF)

S4 Fig Detailed profiles of the features employed by Rhapsody’s random forest classifier. The profiles of the residue-averaged features are plotted in different colors (see legend). On the top panel, the predicted residue-averaged pathogenicity probability is also shown. The dashed vertical lines mark the location of the variants tested experimentally, color-coded with the same scheme used in **Fig 3**.
(TIF)

S5 Fig Scatter plot showing Rhapsody pathogenicity probability distribution for known mutations associated with Bartter syndrome. See **Table 1** for a full list of mutations associated with Bartter syndrome. A variant is classified as “deleterious” if it has a Rhapsody pathogenicity probability higher than 0.5, and is denoted in *red*. In contrast, a mutation incorrectly predicted to be “neutral” by Rhapsody has a probability lower than 0.5. and is depicted by *green* circles. Two variants, T86A and M357T (*yellow circles*), which are associated with Bartter syndrome but are classified as benign by the clinical variant database (see **Table 1**), are correctly predicted as “neutral” by Rhapsody. The graph was made using GraphPad Prism (ver. 8. 1. 2).
(TIF)
