# Supplementary material for: Complementary computational and experimental evaluation of missense variants in the ROMK potassium channel
Source: PLoS Comput Biol. 2020 Apr 6;16(4):e1007749. doi: 10.1371/journal.pcbi.1007749 (PMC7162551; doi:10.1371/journal.pcbi.1007749)
Supplement: S1 Table — The 33 ROMK variants tested experimentally are classified into 4 categories (see last column), based on the measured phenotype: “increased growth” (INC), “no growth defect” (NGD), “modest growth defect” (MGD), “severe growth defect” (SGD). Same color scheme is adopted in Fig 5. “Rhapsody+EVmutation” refers to a combined version of the Rhapsody classifier where EVmutation epistatic score has been included as an additional feature. Computationally predicted classes refer to the clusters of variants defined in Fig 3. (PDF) [file pcbi.1007749.s001.pdf]

Table S1: Experimental results and computational predictions for selected ROMK variants

Variants		Computationally-Predicted Classes (See Figure 3)	Computational Predictions				"Blind" Groups	Exp. Phenotype
Rat	Human		Rhapsody	Rhapsody + EVmutation	EVmutation	PolyPhen-2		
**K80M**	-	pos. ctrl.	del	neu	neu	del	0	**INC (pos. ctrl.)**
**V140M**	-	pos. ctrl.	del	neu	neu	del	0	**INC (pos. ctrl.)**
**H342S**	-	increased fitness	neu	neu	neu	neu	1	**MGD**
**F65L**	-	increased fitness	neu	neu	neu	neu	1	**NGD**
**T86A**	-	increased fitness	neu	neu	neu	neu	1	**NGD**
**A154P**	-	increased fitness	neu	neu	neu	neu	1	**SGD**
**F94L**	-	increased fitness	neu	neu	neu	neu	1	**NGD**
**C358Q**	-	del. (consensus R&P)	del	?	?	del	2	**SGD**
**C355Q**	-	del. (consensus R&P)	del	?	?	del	2	**SGD**
**N361C**	-	del. (consensus R&P)	del	?	?	del	2	**MGD**
**V253D**	-	del. (consensus R&P)	del	?	?	del	2	**MGD**
**N361W**	-	del. (consensus R&P)	del	?	?	del	2	**NGD**
**I85V**	-	neu. (consensus)	neu	neu	neu	neu	3	**NGD**
**V101A**	(A101V)	neu. (consensus)	neu	neu	neu	neu	3	**NGD**
**T247V**	(I247V)	neu. (consensus)	neu	neu	neu	neu	3	**NGD**
**T82L**	-	neu. (consensus)	neu	neu	neu	neu	3	**NGD**
**V105I**	(I105V)	neu. (consensus)	neu	neu	neu	neu	3	**NGD**
**D116A**	(A116D)	neu. (consensus R&P)	neu	?	?	neu	4	**NGD**
**D116S**	(A116S)	neu. (consensus R&P)	neu	?	?	neu	4	**NGD**
**T332S**	-	neu. (consensus R&P)	neu	?	?	neu	4	**NGD**
**Q38R**	-	neu. (consensus R&P)	neu	?	?	neu	4	**NGD**
**L359F**	-	neu. (consensus R&P)	neu	?	?	neu	4	**NGD**
**Y113H**	(H113Y)	neu. (consensus R&P)	neu	?	?	neu	4	**INC (weak)**
**S224C**	-	discordant (R vs. E)	del	p.n	neu	del	5	**NGD**
**Y323H**	-	discordant (R vs. E)	del	p.d	neu	del	5	**NGD**
**K196G**	-	discordant (R vs. E)	neu	p.n	del	neu	5	**MGD**
**T193M**	-	discordant (R vs. E)	del	neu	neu	del	5	**NGD**
**E46N**	(D46N)	discordant (R vs. E)	del	p.d	neu	del	5	**NGD**
**P265R**	-	del. (consensus)	del	del	del	del	6	**SGD**
**P265Y**	-	del. (consensus)	del	del	del	del	6	**MGD**
**C121H**	-	del. (consensus)	del	del	del	del	6	**MGD**
**F291Q**	-	del. (consensus)	del	del	del	del	6	**SGD**
**E318I**	-	del. (consensus)	del	del	del	del	6	**SGD**

The 33 ROMK variants tested experimentally are classified into 4 categories (see last column), based on the *measured phenotype: "increased growth" (INC), "no growth defect" (NGD), "modest growth defect" (MGD), "severe growth defect" (SGD). Same color scheme is adopted in **Fig 5**. "Rhapsody+EVmutation" refers to a combined version of the Rhapsody classifier where EVmutation epistatic score has been included as an additional feature. Computationally predicted classes refer to the clusters of variants defined in **Fig 3.***
