# Supplementary material for: Complementary computational and experimental evaluation of missense variants in the ROMK potassium channel
Source: PLoS Comput Biol. 2020 Apr 6;16(4):e1007749. doi: 10.1371/journal.pcbi.1007749 (PMC7162551; doi:10.1371/journal.pcbi.1007749)
Supplement: S2 Table — Several accuracy estimates over the set of 33 ROMK variants tested experimentally are listed for each method. As explained in the text, there is no unequivocal interpretation of variants displaying “increased growth”. So, we provided measurements obtained by considering them neutral or deleterious, or after excluding them (indicated by ‘-’ label). Since EVmutation and “Rhapsody+EVmutation” classifiers have missing predictions, for the sake of comparison, we also computed accuracy estimates for Rhapsody and PolyPhen-2 on the same subset of variants (*). In parentheses, we show the bootstrapped mean and standard deviations values. (PDF) [file pcbi.1007749.s002.pdf]

Table S2: Accuracy of predictions for 33 ROMK variants tested experimentally, using different metrics

Method	Postulated Function of 'Increased Function' Mutations	Accuracy metric					
		AUROC	AUPRC	MCC	Precision	Recall	F1 Score
Rhapsody	-	0.87 (0.86 ± 0.07)	0.85 (0.85 ± 0.09)	0.46 (0.43 ± 0.16)	0.74 (0.75 ± 0.08)	0.73 (0.72 ± 0.08)	0.74 (0.73 ± 0.08)
Rhapsody	neu.	0.86 (0.86 ± 0.07)	0.83 (0.83 ± 0.10)	0.40 (0.41 ± 0.14)	0.73 (0.74 ± 0.07)	0.70 (0.70 ± 0.07)	0.70 (0.71 ± 0.07)
Rhapsody	del.	0.81 (0.81 ± 0.08)	0.82 (0.81 ± 0.09)	0.45 (0.43 ± 0.14)	0.73 (0.73 ± 0.07)	0.73 (0.71 ± 0.07)	0.73 (0.71 ± 0.07)
PolyPhen-2	-	0.81 (0.80 ± 0.08)	0.70 (0.70 ± 0.11)	0.46 (0.48 ± 0.16)	0.74 (0.77 ± 0.08)	0.73 (0.74 ± 0.08)	0.74 (0.74 ± 0.08)
PolyPhen-2	neu.	0.81 (0.81 ± 0.08)	0.68 (0.69 ± 0.12)	0.40 (0.38 ± 0.17)	0.73 (0.74 ± 0.08)	0.70 (0.69 ± 0.08)	0.70 (0.70 ± 0.08)
PolyPhen-2	del.	0.77 (0.76 ± 0.09)	0.69 (0.70 ± 0.11)	0.45 (0.46 ± 0.18)	0.73 (0.75 ± 0.09)	0.73 (0.73 ± 0.09)	0.73 (0.73 ± 0.09)
EVmutation	-	0.77 (0.79 ± 0.14)	0.85 (0.87 ± 0.11)	0.80 (0.80 ± 0.11)	0.91 (0.92 ± 0.04)	0.90 (0.90 ± 0.06)	0.90 (0.89 ± 0.07)
EVmutation	neu.	0.77 (0.79 ± 0.14)	0.84 (0.86 ± 0.11)	0.81 (0.81 ± 0.11)	0.92 (0.92 ± 0.04)	0.91 (0.91 ± 0.06)	0.91 (0.90 ± 0.07)
EVmutation	del.	0.79 (0.78 ± 0.14)	0.87 (0.85 ± 0.10)	0.67 (0.67 ± 0.13)	0.86 (0.87 ± 0.04)	0.82 (0.82 ± 0.07)	0.81 (0.81 ± 0.08)
Rhapsody +EVmutation	-	0.81 (0.79 ± 0.14)	0.85 (0.84 ± 0.12)	0.47 (0.43 ± 0.22)	0.75 (0.74 ± 0.11)	0.75 (0.73 ± 0.11)	0.75 (0.72 ± 0.11)
Rhapsody +EVmutation	neu.	0.80 (0.79 ± 0.14)	0.83 (0.82 ± 0.14)	0.50 (0.51 ± 0.19)	0.77 (0.79 ± 0.08)	0.77 (0.78 ± 0.08)	0.77 (0.77 ± 0.08)
Rhapsody +EVmutation	del.	0.78 (0.78 ± 0.12)	0.82 (0.83 ± 0.10)	0.36 (0.31 ± 0.19)	0.69 (0.69 ± 0.10)	0.68 (0.66 ± 0.10)	0.67 (0.65 ± 0.10)
Rhapsody*	-	0.84 (0.83 ± 0.09)	0.82 (0.82 ± 0.10)	0.29 (0.29 ± 0.21)	0.66 (0.68 ± 0.11)	0.65 (0.65 ± 0.10)	0.65 (0.65 ± 0.11)
Rhapsody*	neu.	0.81 (0.82 ± 0.08)	0.79 (0.80 ± 0.10)	0.19 (0.17 ± 0.21)	0.63 (0.64 ± 0.11)	0.59 (0.58 ± 0.10)	0.60 (0.59 ± 0.10)
Rhapsody*	del.	0.82 (0.82 ± 0.09)	0.82 (0.83 ± 0.11)	0.37 (0.39 ± 0.20)	0.69 (0.71 ± 0.10)	0.68 (0.70 ± 0.10)	0.68 (0.70 ± 0.10)
PolyPhen-2*	-	0.74 (0.75 ± 0.11)	0.64 (0.65 ± 0.16)	0.29 (0.27 ± 0.23)	0.66 (0.67 ± 0.11)	0.65 (0.65 ± 0.11)	0.65 (0.65 ± 0.11)
PolyPhen-2*	neu.	0.73 (0.74 ± 0.12)	0.61 (0.63 ± 0.15)	0.19 (0.23 ± 0.23)	0.63 (0.67 ± 0.11)	0.59 (0.61 ± 0.11)	0.60 (0.62 ± 0.11)
PolyPhen-2*	del.	0.75 (0.75 ± 0.09)	0.67 (0.69 ± 0.13)	0.37 (0.34 ± 0.20)	0.69 (0.69 ± 0.10)	0.68 (0.67 ± 0.10)	0.68 (0.67 ± 0.10)

Several accuracy estimates over the set of 33 ROMK variants tested experimentally are listed for each method. As explained in the text, there is no unequivocal interpretation of variants displaying “increased growth”. So, we provided measurements obtained by considering them neutral or deleterious, or after excluding them (indicated by ‘-’ label). Since EVmutation and “Rhapsody+EVmutation” classifiers have missing predictions, for the sake of comparison, we also computed accuracy estimates for Rhapsody and PolyPhen-2 on the same subset of variants (*). In parentheses, we show the bootstrapped mean and standard deviations values.

continues below….

Additional accuracy metrics on individual classes (0 = neutral, 1 = deleterious).

Method	Increased Function Labels	Precision (0)	Recall (0)	F1 Score (0)	Precision (1)	Recall (1)	F1 Score (1)
Rhapsody	-	0.81 (0.82 ± 0.10)	0.72 (0.71 ± 0.11)	0.76 (0.75 ± 0.08)	0.64 (0.61 ± 0.13)	0.75 (0.73 ± 0.15)	0.69 (0.66 ± 0.12)
Rhapsody	neu.	0.82 (0.82 ± 0.09)	0.67 (0.68 ± 0.09)	0.74 (0.74 ± 0.07)	0.56 (0.58 ± 0.11)	0.75 (0.75 ± 0.12)	0.64 (0.65 ± 0.10)
Rhapsody	del.	0.76 (0.74 ± 0.10)	0.72 (0.71 ± 0.10)	0.74 (0.72 ± 0.08)	0.69 (0.69 ± 0.10)	0.73 (0.72 ± 0.12)	0.71 (0.69 ± 0.09)
PolyPhen-2	-	0.81 (0.81 ± 0.11)	0.72 (0.73 ± 0.10)	0.76 (0.77 ± 0.08)	0.64 (0.66 ± 0.13)	0.75 (0.76 ± 0.14)	0.69 (0.69 ± 0.11)
PolyPhen-2	neu.	0.82 (0.83 ± 0.10)	0.67 (0.66 ± 0.11)	0.74 (0.73 ± 0.08)	0.56 (0.54 ± 0.13)	0.75 (0.74 ± 0.14)	0.64 (0.62 ± 0.12)
PolyPhen-2	del.	0.76 (0.76 ± 0.10)	0.72 (0.73 ± 0.12)	0.74 (0.74 ± 0.09)	0.69 (0.70 ± 0.13)	0.73 (0.73 ± 0.13)	0.71 (0.71 ± 0.11)
EVmutation	-	0.86 (0.85 ± 0.09)	1.00 (1.00 ± 0.00)	0.92 (0.92 ± 0.06)	1.00 (1.00 ± 0.00)	0.75 (0.75 ± 0.14)	0.86 (0.85 ± 0.10)
EVmutation	neu.	0.88 (0.87 ± 0.09)	1.00 (1.00 ± 0.00)	0.93 (0.93 ± 0.05)	1.00 (1.00 ± 0.00)	0.75 (0.75 ± 0.14)	0.86 (0.85 ± 0.10)
EVmutation	del.	0.75 (0.76 ± 0.09)	1.00 (1.00 ± 0.00)	0.86 (0.86 ± 0.06)	1.00 (1.00 ± 0.00)	0.60 (0.59 ± 0.17)	0.75 (0.73 ± 0.14)
Rhapsody +EVmutation	-	0.77 (0.72 ± 0.14)	0.83 (0.82 ± 0.13)	0.80 (0.76 ± 0.11)	0.71 (0.72 ± 0.18)	0.62 (0.60 ± 0.18)	0.67 (0.64 ± 0.15)
Rhapsody +EVmutation	neu.	0.80 (0.81 ± 0.10)	0.86 (0.87 ± 0.08)	0.83 (0.83 ± 0.07)	0.71 (0.72 ± 0.18)	0.62 (0.63 ± 0.19)	0.67 (0.65 ± 0.16)
Rhapsody +EVmutation	del.	0.67 (0.65 ± 0.13)	0.83 (0.80 ± 0.12)	0.74 (0.71 ± 0.10)	0.71 (0.68 ± 0.19)	0.50 (0.49 ± 0.16)	0.59 (0.55 ± 0.14)
Rhapsody*	-	0.73 (0.71 ± 0.15)	0.67 (0.65 ± 0.14)	0.70 (0.67 ± 0.13)	0.56 (0.57 ± 0.15)	0.62 (0.65 ± 0.17)	0.59 (0.59 ± 0.13)
Rhapsody*	neu.	0.73 (0.72 ± 0.13)	0.57 (0.56 ± 0.13)	0.64 (0.62 ± 0.11)	0.45 (0.44 ± 0.15)	0.62 (0.62 ± 0.18)	0.53 (0.50 ± 0.14)
Rhapsody*	del.	0.73 (0.73 ± 0.12)	0.67 (0.71 ± 0.14)	0.70 (0.71 ± 0.11)	0.64 (0.66 ± 0.16)	0.70 (0.69 ± 0.15)	0.67 (0.66 ± 0.13)
PolyPhen-2*	-	0.73 (0.71 ± 0.14)	0.67 (0.67 ± 0.14)	0.70 (0.68 ± 0.12)	0.56 (0.56 ± 0.17)	0.62 (0.61 ± 0.18)	0.59 (0.57 ± 0.15)
PolyPhen-2*	neu.	0.73 (0.75 ± 0.14)	0.57 (0.59 ± 0.13)	0.64 (0.65 ± 0.11)	0.45 (0.47 ± 0.17)	0.62 (0.65 ± 0.18)	0.53 (0.53 ± 0.15)
PolyPhen-2*	del.	0.73 (0.71 ± 0.15)	0.67 (0.65 ± 0.14)	0.70 (0.67 ± 0.12)	0.64 (0.62 ± 0.14)	0.70 (0.69 ± 0.16)	0.67 (0.64 ± 0.12)
