# Supplementary material for: Complementary computational and experimental evaluation of missense variants in the ROMK potassium channel
Source: PLoS Comput Biol. 2020 Apr 6;16(4):e1007749. doi: 10.1371/journal.pcbi.1007749 (PMC7162551; doi:10.1371/journal.pcbi.1007749)
Supplement: S3 Table — (PDF) [file pcbi.1007749.s003.pdf]

Table S3: Primers used in this study

Primer name	Primer sequence
SmaI_ROMK1_fwd	CATCATCCCGGGATGGGCGCTTCGGAACGGAG
ROMK1_XhoIr_rat	CATCATCTCGAGCTACATCTGGGTGTCGTCCGTTTCATCAACTTC
pTEF1	CAAGTTTCAGTTTCATTTTTCTTGTTCTA
pCYC1	GGACCTAGACTTCAGGTTGTCTA
H342S_f	CGAGTTGATTTTAGTAACTTCGGTAAG
H342S_r	CTTACCGAAGTTACTAAAATCAACTCG
F65L_f	GGTTTATATTCTTAGTGGACATCTGG
F65L_r	CCAGATGTCCACTAAGAATATAAACC
T86A_f	CGTGTTCATCGCAGCCTTCTTG
T86A_r	CAAGAAGGCTGCGATGAACACG
A154P_f	CAGAACAGTGCCCTACTGCCATTTTC
A154P_r	GAAAATGGCAGTAGGGCACTGTTCTG
F94L_f	GAGTTGGTTCTTCTTTGGTCTC
F94L_r	GAGACCAAAGAAGAACCAACTC
C358Q_f	CTGTGCCATGCAGCTCTATAATG
C358Q_r	CATTATAGAGCTGCATGGCACAG
C355Q_f	AGACCCCTCACCAGGCCATGTGCCT
C355Q_r	AGGCACATGGCCTGGTGAGGGGTCT
N361C_f	GTGCCTCTATTGTGAGAAAGATG
N361C_r	CATCTTTCTCACAATAGAGGCAC
V253D_f	AACTTTGTCGACGACGCTGGC
V253D_r	GCCAGCGTCGTCGACAAAGTT
N361W_f	GTGCCTCTATTGGGAGAAAGATGC
N361W_r	GCATCTTTCTCCCAATAGAGGCAC
I85V_f	TGACCGTGTTCGTTACAGCCTTCTT
I85V_r	AAGAAGGCTGTAACGAACACGGTCA
I247V_f	CATCATTTTGGATCAGGTTAACATCAACTTTGTCG
I247V_r	CGACAAAGTTGATGTTAACCTGATCCAAAATGATG
T82L_f	GGTACAAAATGCTTGTGTTCATCAC
T82L_r	GTGATGAACACAAGCATTTTGTACC
V105I_f	CGTAGCGTATATTCATAAGGAC
V105I_r	GTCCTTATGAATATACGCTACG
D116A_f	TACCCGCCTGCTAACCGCACTC
D116A_r	GAGTGCGGTTAGCAGGCGGGTA
A116S_f	CTACCCGCCTTCTAACCGCA
A116S_r	TGCGGTTAGAAGGCGGGTAG
T332S_f	TGTGTCCAAGTCCAAGGAAGG
T332S_r	CCTTCCTTGGACTTGGACACA
Q38R_f	CGTTCCCGGAGACGGGCAAG
Q38R_r	CTTGCCCGTCTCCGGGAACG
L359F_f	GTGCCATGTGCTTCTATAATGAG
L359F_r	CTCATTATAGAAGCACATGGCAC
Y113H_f	CCCAGAGTTCCATCCGCCTGACAA
Y113H_r	TTGTCAGGCGGATGGAACTCTGGG
S224C_f	CTTCTGATTGGCTGTCACATATATGG
S224C_r	CCATATATGTGACAGCCAATCAGAAG
Y323H_f	GCTTTGGGGCCATCGTTTCGTTC
Y323H_r	GAACGAAACGATGGCCCCAAAGC
K196G_f	CATTACGTTCAGCGGTAATGCGGTGATC
K196G_r	GATCACCGCATTACCGCTGAACGTAATG
T193M_f	GCTAAAACCATTATGTTCAGCAAGAATG
T193M_r	CATTCTTGCTGAACATAATGGTTTTAGC
E46N_f	CTGGTCTCTAAAAATGGAAGATGTAAC
E46N_r	GTTACATCTTCCATTTTTAGAGACCAG
P265R_f	CTTCATATCCAGACTGACGATC
P265R_r	GATCGTCAGTCTGGATATGAAG
P265Y_f	GTTCTTCATATCCTATCTGACGATCTACC
P265Y_r	GGTAGATCGTCAGATAGGATATGAAGAAC
C121H_f	ACCGCACTCCTCACGTGGAGAACA
C121H_r	TGTTCTCCACGTGAGGAGTGCGGT
F291Q_f	CCAACAGGACCAAGAGCTGGTGGT
F291Q_r	ACCACCAGCTCTTGGTCCTGTTGG
E318I_f	CGTCCCAGAGATAGTGCTTTGGG
E318I_r	CCCAAAGCACTATCTCTGGGACG
