# Supplementary figures and images for: Complementary computational and experimental evaluation of missense variants in the ROMK potassium channel
Source: PLoS Comput Biol. 2020 Apr 6;16(4):e1007749. doi: 10.1371/journal.pcbi.1007749 (PMC7162551; doi:10.1371/journal.pcbi.1007749)

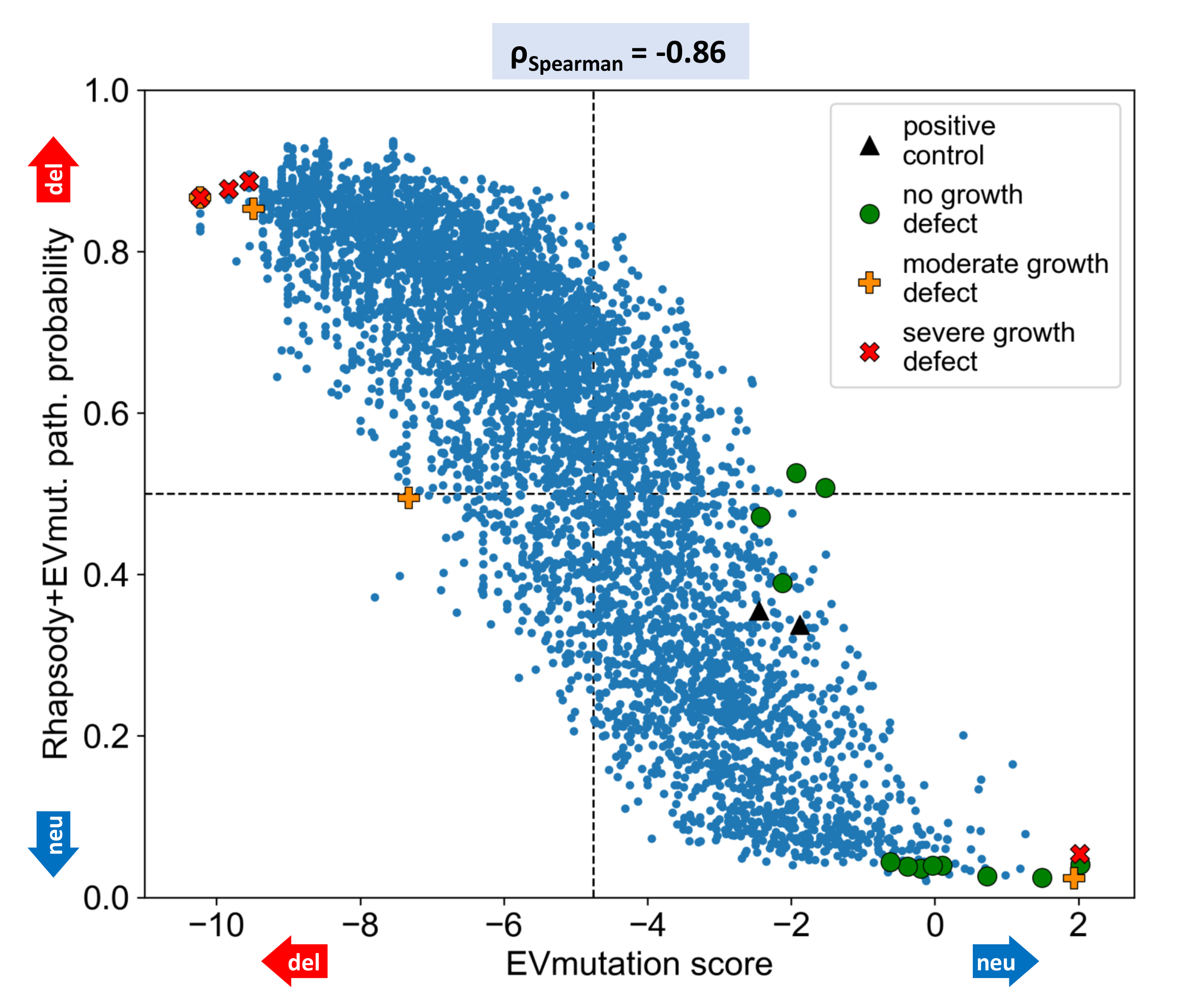

Supplement: S1 Fig — This figure has the same format as Fig 3, but the y-axis is replaced by predictions from the combined Rhapsody classification scheme that incorporates EVmutation’s epistatic scores directly into the Random Forest training algorithm of the classifier, as an additional feature. See Materials and Methods for additional information. (TIF) [file pcbi.1007749.s004.tif]

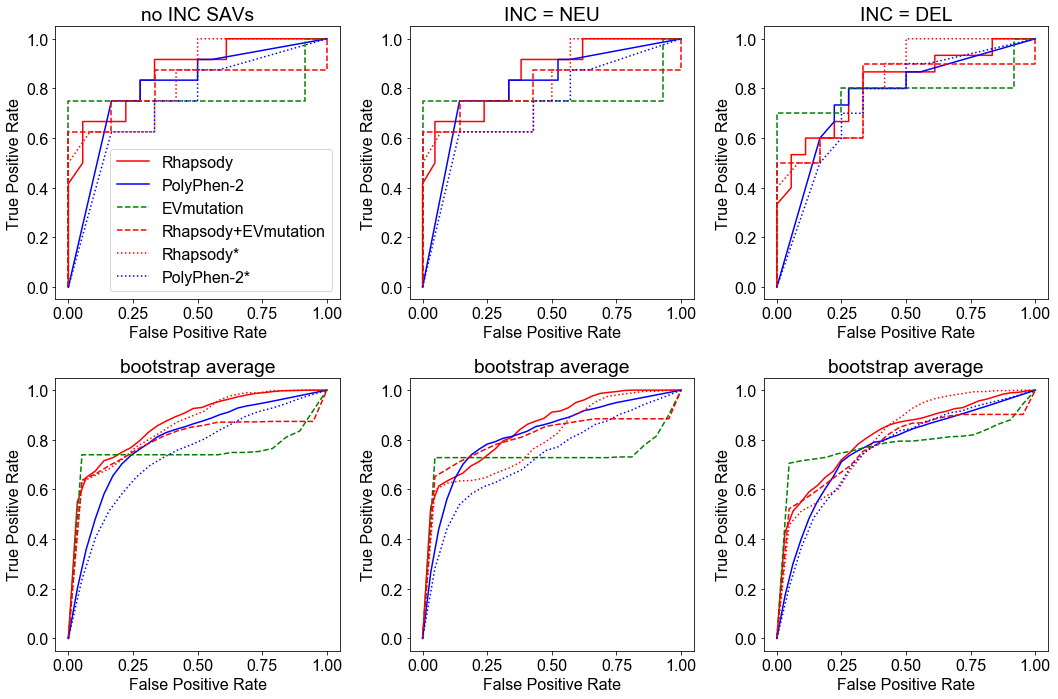

Supplement: S2 Fig — EVmutation and “Rhapsody+EVmutation” classifiers (dashed lines) only return predictions for 22 out of 33 variants. For the sake of comparison, as in S2 Table, we also show ROC curves for Rhapsody and PolyPhen-2 predictions on the same subset of variants (*, dotted lines). Variants displaying “increased growth” (INC) phenotype have been excluded (left column) or included as “neutral” or “deleterious” (center and right columns, respectively). (TIF) [file pcbi.1007749.s005.tif]

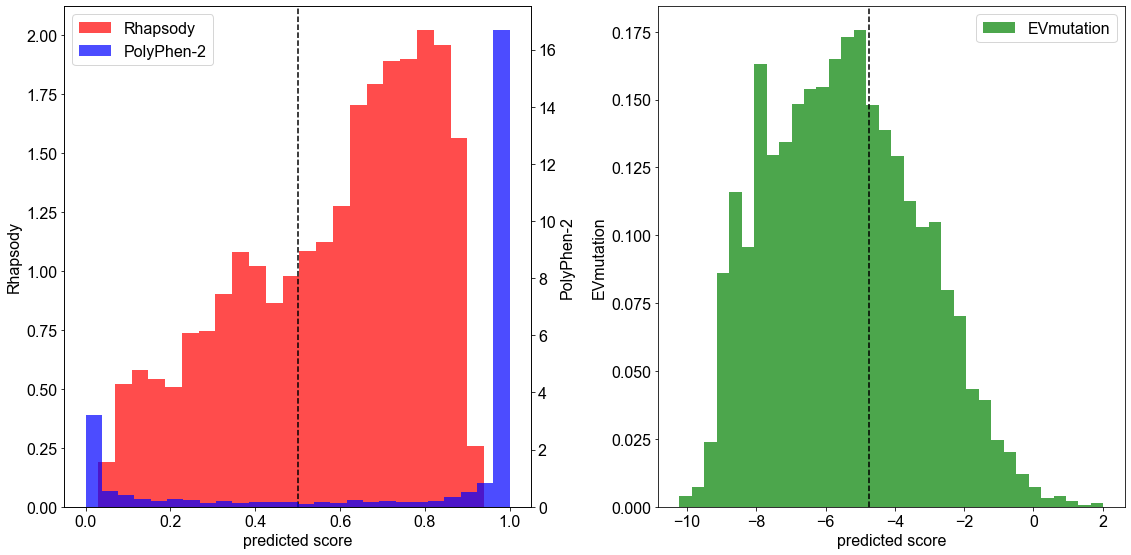

Supplement: S3 Fig — Normalized histograms are displayed using the data presented in Figs 2 and 3. (TIF) [file pcbi.1007749.s006.tif]

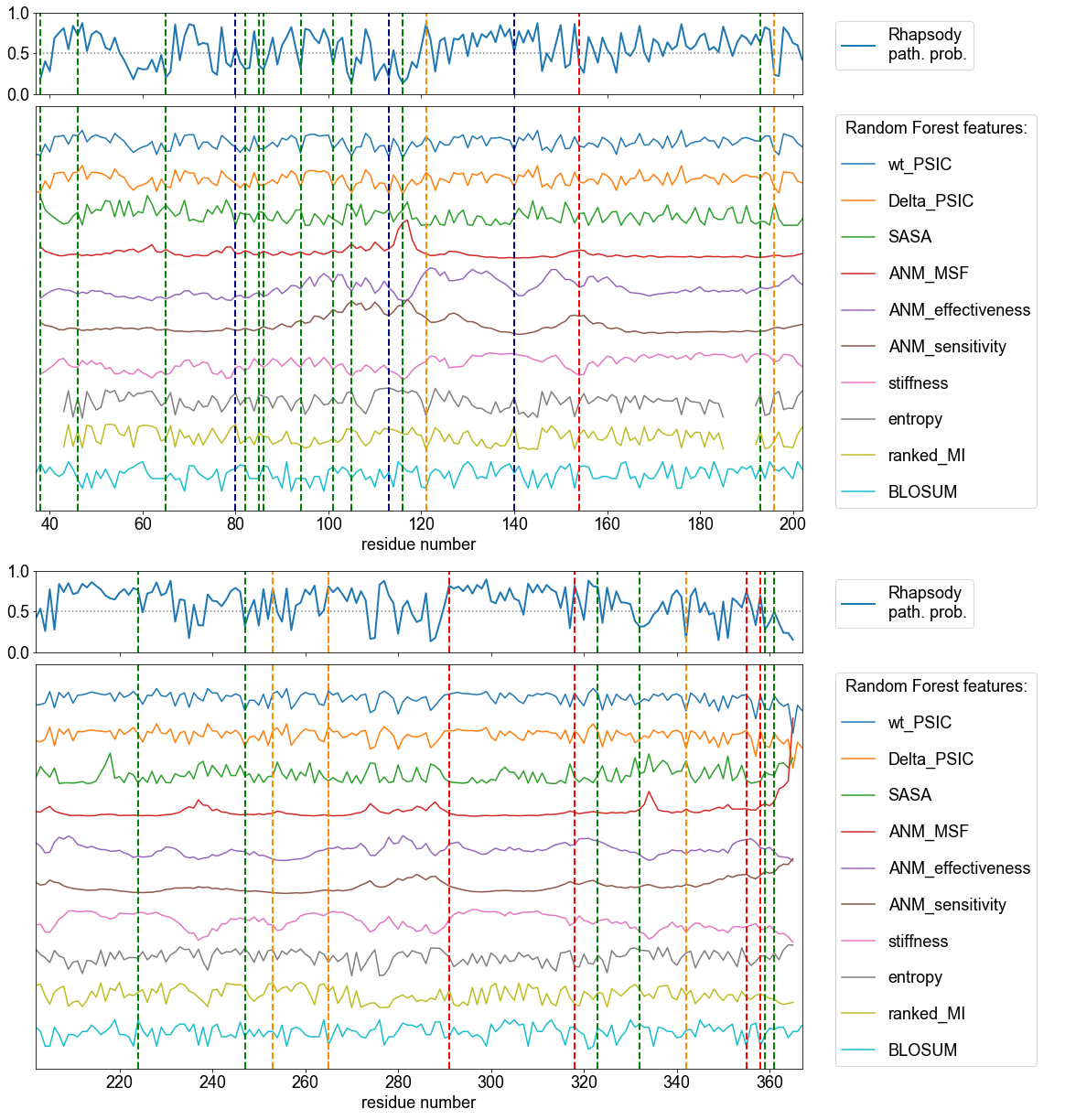

Supplement: S4 Fig — The profiles of the residue-averaged features are plotted in different colors (see legend). On the top panel, the predicted residue-averaged pathogenicity probability is also shown. The dashed vertical lines mark the location of the variants tested experimentally, color-coded with the same scheme used in Fig 3. (TIF) [file pcbi.1007749.s007.tif]

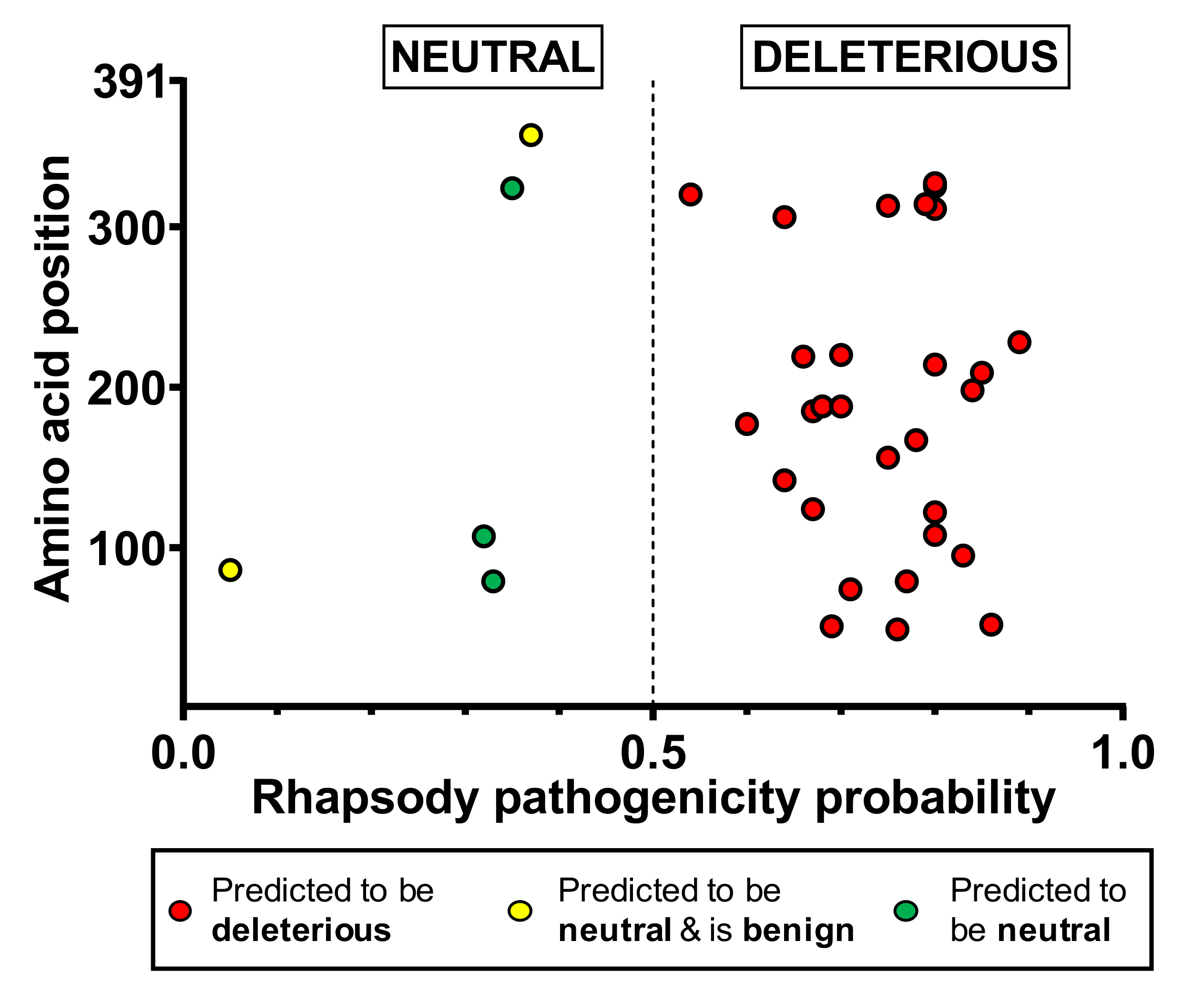

Supplement: S5 Fig — See Table 1 for a full list of mutations associated with Bartter syndrome. A variant is classified as “deleterious” if it has a Rhapsody pathogenicity probability higher than 0.5, and is denoted in red. In contrast, a mutation incorrectly predicted to be “neutral” by Rhapsody has a probability lower than 0.5. and is depicted by green circles. Two variants, T86A and M357T (yellow circles), which are associated with Bartter syndrome but are classified as benign by the clinical variant database (see Table 1), are correctly predicted as “neutral” by Rhapsody. The graph was made using GraphPad Prism (ver. 8. 1. 2). (TIF) [file pcbi.1007749.s008.tif]
